# Emerging Role of Sphingosine-1-phosphate in Inflammation, Cancer, and Lymphangiogenesis

**DOI:** 10.3390/biom3030408

**Published:** 2013-07-23

**Authors:** Wei-Ching Huang, Masayuki Nagahashi, Krista P. Terracina, Kazuaki Takabe

**Affiliations:** Division of Surgical Oncology, Department of Surgery, Virginia Commonwealth University School of Medicine and Massey Cancer Center, Richmond, VA 23298-0011, USA; E-Mails: wchuang@vcu.edu (W.-C.H.); mnagahashi@vcu.edu (M.N.); kmpennington@mcvh-vcu.edu (K.P.T.)

**Keywords:** sphingolipids, sphingosine-1-phosphate, inflammation, lymphangiogenesis, cancer, lymphatic endothelial cell, VEGF, angiopoietin, spinster 2, metastasis

## Abstract

The main function of the lymphatic system is to control and maintain fluid homeostasis, lipid transport, and immune cell trafficking. In recent years, the pathological roles of lymphangiogenesis, the generation of new lymphatic vessels from preexisting ones, in inflammatory diseases and cancer progression are beginning to be elucidated. Sphingosine-1-phosphate (S1P), a bioactive lipid, mediates multiple cellular events, such as cell proliferation, differentiation, and trafficking, and is now known as an important mediator of inflammation and cancer. In this review, we will discuss recent findings showing the emerging role of S1P in lymphangiogenesis, in inflammation, and in cancer.

## 1. Introduction

The lymphatic system, composed of lymphatic vessels and lymphatic fluid, exerts important biological functions such as drainage of interstitial fluids and proteins to the blood stream and the transport of immune cells and nutrients [1]. Lymphangiogenesis is the process of formation of new lymphatic vessels from existing ones. Multiple signaling pathways orchestrate proliferation, sprouting and migration of lymphatic endothelial cells (LECs). Vascular endothelial growth factors (VEGF)-C and VEGF-D and their receptor (VEGFR-3) are the most studied proteins in lymphangiogenesis. In adulthood, lymphangiogenesis can occur when LECs are stimulated by inflammation- or tumor-associated factors. Sphingosine-1-phosphate (S1P), which is also upregulated in inflammation and in tumors, has recently gained attention as a mediator involved in lymphangiogenesis.

## 2. Lymphatic System

The circulatory system transports blood and lymphatic fluid in our body. Lymphatic fluid is defined as the fluid component within lymphatic vessels, containing interstitial fluid, macromolecules, and cells collected from the blood vascular system by lymphatic capillaries [2]. The lymphatic vasculature forms a unidirectional network commencing from peripheral tissue as blind-ended capillaries which then merge to pre-collecting vessels, collecting vessels, and finally, to afferent vessels to lymph nodes [3]. Lymphatic capillaries are composed of a thin-layer of oak leaf-shaped LECs, which have discontinuous button-like cell-cell junctions [4]. These intrajunctional gaps provide highly permeable sites for leukocyte entry and uptake of lymphatic fluid components. The main functions of the lymphatic vessel network are maintaining tissue fluid homeostasis, transporting lipids and nutrients, and immune cell trafficking [1]. Lymphatic vessels drain interstitial fluids from peripheral tissue into the thoracic duct, returning the fluid to the blood circulation and providing routes for immune cell trafficking [5,6]. The anatomy of lymphatic vasculature implies an important function for this system in immune cell trafficking. Abundance of lymphatic vessels in skin, airway, and gut—the sites frequently exposed to pathogens or environmental factors—indicates that the lymphatic system participates in the regulation of inflammatory responses through its role as the trafficking route for lymphocyte transport to the lymph nodes for immune surveillance [5]. Failure of lymphatic system function can cause lymphedema, excessive fluid accumulation in tissue, and alteration of immune responses [3,7].

## 3. Regulators of Lymphangiogenesis

Lymphangiogenesis is defined as the formation of new lymphatic vessels from existing ones. It occurs during embryonic development and becomes stable and quiescent after establishment of the lymphatic vasculature [3]. However, lymphangiogenesis can also be stimulated in adulthood to play physiological or pathological functions. It is known to occur in response to inflammatory conditions in peripheral tissues (skin, airway, and gut) and diseases such as inflammatory bowel diseases, rheumatoid arthritis, and cancer [8,9,10,11]. The regulation of lymphangiogenesis includes molecular mechanisms that are primarily mediated by growth factor-growth factor receptor systems (Table 1) and cellular mechanisms, predominantly involving myeloid cells. 

**Table 1:** Growth factors involved in lymphangiogenesis.

Growth factor	Receptor	Main function
**VEGF-A **	VEGFR-2	● Recruit macrophage to facilitate inflammatory lymphangiogenesis ● Generate giant and abnormal lymphatic vessels in cancer and chronic inflammation
**VEGF-C **	VEGFR-2* VEGFR-3 Neuropilin-2	● Mediate embryonic lymphatic development ● Induce proliferation, migration, and survival of lymphatic endothelial cells in inflammation and cancer
**VEGF-D **	VEGFR-2* VEGFR-3 Neuropilin-2	● Induce proliferation, migration, and survival of lymphatic endothelial cells in inflammation and cancer
**Angiopoietin-1 **	Tie2	● Mediate post-natal lymphatic patterning
**Angiopoietin-2 **	Tie2	● Mediate post-natal lymphatic patterning ● Might contribute to tumor-induced lymphangiogenesis

* After proteolytical processing, mature forms of VEGF-C and VEGF-D could bind to VEGFR-2.

### 3.1. Vascular Endothelial Growth Factor (VEGF)/VEGFR System

The discovery of vascular endothelial growth factors (VEGFs) and their receptors (VEGFRs) stemmed from angiogenesis research [12,13]. The mammalian VEGF family includes five members: VEGF (also called VEGF-A), placenta growth factor (PIGF), VEGF-B, VEGF-C, and VEGF-D. The VEGFR family includes VEGFR-1, VEGFR-2, and VEGFR-3. Typical signaling from VEGFRs promote cell survival, proliferation, and migration [14]. Among them, VEGF-C, VEGF-D, and their receptor VEGFR-3 are key regulators of lymphangiogenesis in both physiological and pathological settings and provided the first insights into how lymphangiogenesis occurs [7,15,16,17,18,19,20,21]. Overexpression of either VEGF-C or VEGF-D causes lymphangiogenesis; however, only VEGF-C is involved in embryonic lymphatic development [22,23,24,25,26,27,28]. VEGF-C expression is found at the sites of lymphatic sac formation in development as well as in vascular smooth muscles cells and lymph nodes in adults [25,29,30,31], while VEGF-D is only expressed in adult tissue such as the lungs, heart, skeletal muscle, and intestine [32].

A unique two-step proteolytic cleavage enables VEGF-C and VEGF-D precursor proteins to convert to active and mature forms and therefore determines their binding affinity to VEGFR-2 or VEGFR-3 [33,34]. Only fully processed mature forms can bind to VEGFR-2. The role of VEGFR-3 in lymphangiogenesis has been established in VEGFR-3-deficient mice, which have been shown to have a similar phenotype to VEGF-C-deficient mice [25,27]. In addition to VEGFRs, VEGF-C and VEGF-D also bind to neuropilin-2 (Nrp2), a semaphorin receptor in the nervous system that is also expressed in lymphatic capillaries [23]. Binding of Nrp2 modulates VEGFRs signaling by providing specificity of signal transduction [35,36,37]. Consistently, Nrp2-deficient mice have lymphatic hypoplasia [38]. Other than VEGF-C and VEGF-D, VEGF-A has also been shown to induce lymphangiogenesis in cancer [39] and chronically-inflamed tissue [40]. VEGF-A binds to VEGFR-2 to activate lymphangiogenesis; however, VEGF-A still cannot replace the role of VEGF-C in embryonic lymphatic development [25,41]. These reports strengthen the central role of VEGF-C in developmental lymphangiogenesis.

### 3.2. Angiopoietin/Tie System

In addition to the VEGF/VEGFR system, angiopoietins (Ang1, Ang2, and Ang3/4) and their receptors (Tie1 and Tie2) are also known as mediators for vascular vessel remodeling and integrity [42]. Tie1 and Tie2 are the only known endothelial cell-specific receptor tyrosine kinases [43,44]. Tie1-deficient mice showed compromised integrity of vessel endothelial cells and pulmonary edema and died in utero [45]. Mice with deficiency or loss-of-function Tie2 died in embryo due to inability to expand the vasculature system [46,47]. Ang1, Ang2, and Ang3/Ang4 have high sequence homology and all have been reported to promote lymphangiogenic sprouting [48,49,50]. Although all four of the angiopoietins have been shown to interact with Tie2 [51,52,53], Ang1 and Ang2 are the best characterized compared to the others to date. Ang1 is an obligate agonist of Tie2 receptor while Ang2 acts both as agonistic and as antagonistic in dose- and context-dependent manners [51,52,54]. Ang2 is persistently expressed in LECs, whereas Ang1 is downregulated by prospero-related homeodomain transcription factor (Prox-1) when blood vessel-derived endothelium is reprogrammed to lymphatic endothelium by Prox-1 [55,56]. In addition, Ang-2-deficient mice have shown lymphatic vasculature defects that can be rescued by Ang1 [54,57]. More studies are needed to further delineate the complexity of the interrelationship between Ang1 and Ang2.

### 3.3. Myeloid Cells

Myeloid cells including leukocytes and macrophages also have been shown to participate in lymphangiogenesis [58]. A role for macrophages in post-natal lymphatic vessels remodeling has been suggested in mice deficient of macrophage colony stimulatory factor (M-CSF), where lymphatic vessel branching is reduced due to absence of macrophages in these mice [8]. In the adulthood of these mice, however, no abnormality can be observed. B cells also regulate expansion of lymphatic system as observed in lymphocyte-deficient mice [59,60]. In response to inflammatory stimuli such as lipopolysaccharide (LPS) or TNF-α, infiltrated macrophages express more VEGF-C and VEGF-D while LECs express higher Prox-1 and NF-κB to upregulate VEGFR-3 expression [61,62]. In a high-salt-diet-induced hypertension model, macrophages and dendritic cells are found to regulate the expansion of the lymphatic capillary network in skin through providing VEGF-C [63]. In various mouse tumor models, macrophage recruitment has been demonstrated to promote lymphangiogenesis [64,65,66,67,68]. Tumor-associated macrophages have also been linked to increased peri-tumoral lymphangiogenesis and metastasis in human cancer such as breast cancer [69], cervical cancer [65], squamous cell carcinoma [70], and advanced colorectal cancer [71]. Although still in debate, the general mechanism by which these macrophages, B cells, and dendritic cells contribute to lymphatic remodeling is through providing lymphangiogenic factors such as VEGF-A, VEGF-C, and VEGF-D.

## 4. Lymphangiogenesis during Development

Lymphatic vascular development involves differentiation of LECs, lymphangiogenesis, and remodeling [3]. The blood vascular system develops earliest in embryos. Blood endothelial cells (BECs), differentiated from hemangioblast progenitors, form the primitive vascular plexus that is then remodeled into a vascular network. The first LECs are differentiated from a subpopulation of BECs in the cardinal vein and then sprout out to form primitive lymphatic sacs in regions where lymphangiogenic VEGF-C is expressed [3,25,72,73,74]. The distinct terminal differentiation between LECs and BECs enables the discovery of lymphatic vascular-specific markers such as Prox1, the membrane glycoprotein podoplanin (D2-40), VEGFR-3, and lymphatic vessel hyaluronan receptor-1 (LYVE-1) [7,75,76,77,78]. These main transcription factors, Sox18, COUP-TFII and Prox1, orchestrate LEC differentiation [72,79,80,81,82,83].

Once lymphatic sacs are formed, lymphatic vessels sprout from lymph sacs and then are remodeled into the lymphatic vascular network [6,76]. After embryonic development, functional lymphatic vasculature is established and quiescent. However, physical lymphangiogenesis in adults can occur in certain conditions, such as immunity [10,60,84,85,86], during wound healing [87,88], and at the sites of transplanted tissue [89,90,91]. Lymphangiogenesis helps to decrease inflammation-induced edema, and to transport extravasated leukocyte and antigen presenting cells from inflamed tissue to lymphoid organs to initiate specific immune responses [2,5].

## 5. Lymphangiogenesis in Inflammation

Although lymphangiogenesis is restricted to the site of inflamed tissue and wound healing in adulthood, pathological lymphangiogenesis also happens in the setting of dysregulated inflammatory responses or when cancer cells take advantage of lymphangiogenesis to facilitate their progression [6,92,93,94,95,96,97,98,99].

Lymphangiogenesis can be observed in inflamed peripheral tissue and its draining lymph nodes. Similar as in embryonic development, the VEGF-C/VEGFR-3 axis has been identified as a key mediator of inflammation-driven lymphangiogenesis as described in the model of cornea neovascularization [100]. In a mouse airway infection model, VEGF-C and VEGF-D-expressing immune cells drive lymphangiogenesis. Another mouse peritonitis model showed inflammatory lymphangiogenesis is triggered through NF-κB-mediated upregulation of Prox-1 and VEGFR-3 [62]. Inflammation promotes lymphatic vessel growth by pro-inflammatory cytokines like TNF-α and IL-1β which upregulate VEGF-C though NF-κB-mediated promoter activation [101]. The relationship between lymphangiogenesis and inflammation is somewhat reciprocal. Lymphangiogenesis has a physical role in the clearance of fluid and infiltrated inflammatory mediators to resolve inflammation as it does in tissue repair and wound healing; however, pervasive lymphangiogenesis in response to overexpressed VEGF-C or VEGF-A could delay lymphatic fluid clearance as well [41,102]. It has been observed that once lymphangiogenesis has been established during inflammation, the newly formed lymphatic vessels persist for months even when the inflammation has been resolved [8]. This might be an important clue for the pathological involvement of lymphangiogenesis in chronic inflammatory diseases. 

In addition to the VEGF-C/VEGFR-3 axis, VEGF-A expression is also upregulated in certain inflammatory circumstances such as rheumatoid arthritis and delayed-type hypersensitivity; however, VEGF-A and VEGFR1/2 seem to participate in lymphangiogenesis in a more context-dependent way. In a TNF-α-induced rheumatoid arthritis model, lymphangiogenesis was reduced by neutralization of VEGFR-2 [103]. In a delayed-type hypersensitivity model, which is induced in the ear skin of transgenic mice that overexpress VEGF-A specifically in the epidermis [9], the inflammatory lesions displayed promoted lymphatic vessel proliferation and enlargement, which might contribute to prolonged inflammatory responses, by blocking both VEGFR-1 and VEGFR-2 suppressed lymphangiogenesis as well as inflammation. Nonetheless, no significant effect on lymphangiogenesis was observed by inhibiting either VEGFR-1 or VEGFR-2 in a bacterial infection-induced chronic airway inflammation model [8].

The involvement of myeloid cells in inflammatory lymphangiogenesis is also commonly observed. For example, B cells are found to drive lymphangiogenesis in inflamed lymph nodes [60]. In a murine corneal inflammation model, a rapid increase of VEGFR-3 and VEGF-C expressing dendritic cells are found in the cornea [104]. Macrophages are recruited to inflamed corneas in response to VEGF-A, and to release VEGF-C and VEGF-D to mediate inflammatory lymphangiogenesis in a rabbit corneal inflammation model [100].

## 6. Lymphangiogenesis in Cancer

Tumor-associated lymphangiogenesis has been observed and implicated in cancer progression by its role of providing routes for immune cell recruitment and for cancer metastasis [92,105,106]. It has been shown that tumor-associated lymphangiogenesis usually causes formation of abnormal and leaky lymphatic vessels, which facilitates access for cancer cells to metastasize [107,108]. As in developmental lymphangiogenesis, transcription factors Sox18 and COUP-TFII and the VEGF-C/VEGFR-3/Nrp2 system are utilized by tumor cells to regulate LEC differentiation and lymphatic vessel sprouting, respectively. Usually, the expression of Sox18 and COUP-TFII is suppressed after development; however, studies have indicated that these proteins might be re-expressed by tumor cells to facilitate lymphangiogenesis and metastasis. In experimental tumor models, Sox18 deficiency or COUP-TFII inactivation has been shown to cause reduced tumor lymphangiogenesis and metastasis [109,110]. COUP-TFII might exert its lymphangiogenic function through Nrp2-increased VEGF-C signaling [109].

Studies have demonstrated that VEGF-C and VEGF-D induce lymphangiogenesis, lymphatic invasion, and nodal metastasis in various experimental tumor models [92,111,112,113,114,115,116,117,118,119]. Collaborative action of VEGF-C and another growth factor, fibroblast growth factor-2 (FGF-2), has also been reported [120]. Reciprocally, blocking VEGFR-3, Nrp2, or FGF-2 has been shown to inhibit tumor growth, lymphaniogensis, and metastasis [37,115,121,122].

Early stage of tumorigenesis requires involvement of Ang2 as demonstrated in Ang2-deficient mice [123]. Moreover, Ang2/Tie2 signaling has been implicated in tumor-induced angiogenesis and tumor growth and metastasis [124,125]. Further studies will be needed to further delineate the contribution of the Ang2/Tie2 system in tumor-induced lymphangiogenesis. The involvement of myeloid cells in tumor-induced angiogenesis is still a matter of debate. Some evidence exists for trans-differentiation of hematopoietic cell-derived endothelial progenitors to leukocytes and macrophages precipitating the process of growing vessels in tumor [58], while other reports show no evidence for the contribution of either bone marrow-derived cells or macrophages [126,127].

## 7. Sphingosine-1-phosphate (S1P)

Sphingosine-1-phosphate (S1P) is a bioactive lipid involved in a broad spectrum of cellular process such as cell survival, proliferation, differentiation, migration, and trafficking [128,129]. S1P is formed in cells by phosphorylation of sphingosine by sphingosine kinases (SphK1 and SphK2). Breakdown of S1P can be achieved by irreversible hydrolysis by S1P lyase or reversible dephosphorylation by S1P phosphatases (SPP1 and SPP2) back to sphingosine [130]. Intracellular S1P is a second messenger to trigger calcium release from the endoplasmic reticulum [131,132,133]. Important intracellular target proteins of S1P such as histone deacetylases (HDACs) and tumor necrosis factor (TNF)-associated factor 2 (TRAF2) have been identified [134,135]. These findings further address the molecular mechanisms by which S1P mediates TNF-α signaling and epigenetic regulation.

Intracellular S1P can be exported by several transporters, such as the ATP-binding cassette transporters ABCA1 [136], ABCC1 [137,138], ABCG2 [138], and Spinster 2 (Spns2) [139,140,141,142,143,144,145,146,147]. Interestingly, ABCC1 and ABCG2 were originally identified as multi-drug resistant genes [138], and correlate with worse prognosis in breast cancer [148]. Discovery of these S1P transporters explains the diverse autocrine and paracrine actions of S1P. The “inside-out signaling” of S1P is termed to describe that activation of SphK1 produces S1P which is exported and then binds to five specific G-protein-coupled receptors, S1PRs (S1PR1-5) [129]. The combination and cell-type-specific expression of different S1PRs determines a broad range of biological functions mediated by S1P [129,149,150]. Owing to its multiple biological functions, S1P is implicated in various physiological and pathological conditions such as inflammation and cancer [128,150,151].

## 8. S1P in Inflammation

S1P is now emerging as an important mediator of multiple aspects of both innate and adaptive immunity [149,150]. One of the most important functions of S1P is regulation of immune cell trafficking. Concentration of S1P in the blood is much higher than within the tissue, and this S1P gradient is important for lymphocyte trafficking [150,152]. Lymphocytes sense this S1P gradient, and by altering S1PRs expression, egress from lymphoid organs to the blood [153,154,155,156]. Ample evidence of S1P’s role has been collected in experiments using mice with genetic loss of S1PR1 [157,158] as well as in models that have downregulation of S1PR1 by using a functional antagonist of S1PR1, FTY720, which is an FDA-approved drug for treatment of multiple sclerosis [159]. FTY720 is a structural analog of S1P that will be phosphorylated by SphK1 or SphK2 and produce phosphorylated-FTY720 (FTY720-P) [160,161,162]. FTY720-P binds to all the S1PRs except for S1P_2,_ acting as a functional antagonist. FTY720-P induces internalization and degradation of the S1PR1 therefore affecting lymphocyte trafficking by decreasing the number of mature circulating lymphocytes and preventing lymphocyte egress from lymphoid organs [163,164,165]. This unique action is what makes FTY720 an immunosuppressive agent for treating the autoimmune disease multiple sclerosis. Preclinical evidence is still accumulating in other inflammatory disease such as colitis [166], arthritis [167], and asthma [168]. 

Furthermore, the intracellular actions of S1P also play important role in inflammation by activation of the transcription factor NF-κB, which is required in inflammatory and immune responses [134,169]. S1P has been shown to mimic the effect of the inflammatory cytokine TNF-α to activate endothelial cell activation through NF-κB [170]; subsequently, the biological action of TNF-α has been shown to occur through activation of SphK1. Furthermore, SphK1 has proven to be an indispensable mediator in LPS, TNF-α, and IL-β signaling and pro-inflammatory function [171,172,173]. 

## 9. S1P in Cancer Progression

The role of S1P in cancer progression has been established by studies demonstrating that the upregulation/activation of SphK1 and production of S1P inhibits apoptosis and facilitates survival of cancer cells, thus promoting tumor growth, angiogenesis, and metastasis [151,174]. Numerous studies reveal the oncogenic role of SphK1; however, the isoform SphK2 seems to possess not only an overlapping role with SphK1 in promoting tumor development but also an opposing role in inducing apoptosis [175,176]. The mechanism by which SphK2-produced S1P acts as an endogenous HDAC inhibitor [135] might suggest a more sophisticated role of SphK2 in cancer progression due to the varied contexts of epigenetic regulation among different cell types. In solid tumors, SphK1 is required in the oncogenic signaling of VEGF, epidermal growth factor (EGF), and Ras [177,178,179]. Overexpression of SphK1 has been identified in mRNA screening or immunohistochemistry staining in multiple cancer cells derived from breast, colon, lung, ovary, stomach, uterus, kidney, and rectum [180,181,182]. Inhibition of SphK1 with its specific inhibitor SK1-I reduces the growth of acute myelogenous leukemia and glioblastoma [183,184]. A recent study has proposed a new SphK2 specific inhibitor ABC294640 [185] which reduces S1P levels and inhibits cancer cells proliferation *in vitro* and *in vivo*, and might be used to further dissect the biological functions between the two isoforms. 

As discussed above, cancer cells may adapt both the intracellular actions of S1P and inside-out signaling of S1P to promote their survival and metastasis. S1P may act on intracellular targets such as HDACs and NF-κB to promote cancer progression [134,135]. Tumor cells export S1P to act through S1PRs to promote growth, survival, motility and metastasis in an autocrine manner [186,187]. A paracrine action of tumor cells-exported-S1P is to induce the production of endothelial adhesion molecules, angiogenesis, and to regulate tumor–stromal interactions as well as immune cells [188].

S1PR1 has been shown to mediate persistent activation of signal transducer and activator of transcription-3 (STAT3) in tumor. Activated STAT3 therefore plays two regulatory roles as transcription factor for both S1PR1 and IL-6, which is the most potent oncogenic cytokine [189]. In agreement with this report, our recent study further demonstrates the SphK1/S1P/S1PR1 axis links STAT3 and NF-κB persistent activation in colitis-associated colon cancer [190].

## 10. Role of S1P in Lymphangiogenesis

The role of S1P in determining the fate of vascular cells is much known, mainly through interaction of S1PRs, which are coupled with different combinations of G proteins. S1PR1 couples with the Gi protein family, while S1PR2 and S1PR3 couple to the G_i_, G_q_, and G_12/13_ protein families. S1P regulates vascular endothelial cell proliferation, migration, and morphogenesis. S1PR1-deficient mice have been shown to have incomplete vascular maturation, dying in embryo due to hemorrhage [157]. S1PR1-mutant cells have shown an inability to activate the small GTPase, Rac, therefore leading to a defective migration response. Attempts to block S1PRs by FTY720 or extracellular S1P by anti-S1P-neutralizing antibody have resulted in an inhibition of tumor-induced angiogenesis [191,192]. Together, this evidence highlights the essential role of S1PR1 and S1P signaling in blood vessel formation and mammalian development. Regarding the close similarity of regulation and intimate crosstalk of angiogenesis and lymphangiogenesis, a role for S1P in lymphangiogenesis has been expected [193].

**Figure 1:**
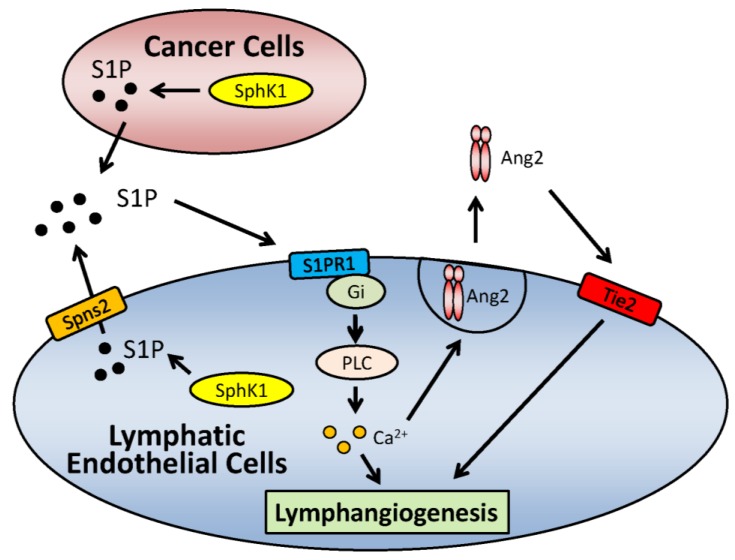
Sphingosine-1-phosphate (S1P) in cancer-induced lymphangiogenesis.

Following from the various overlapping roles of S1P and lymphangiogenesis in physical and pathological contexts, studies to address their interrelationship have been accumulating during the past five years [194]. As summarized in Figure 1, Yoon *et al.* first depicted the elegant signaling pathways by which S1P promotes lymphangiogenesis via an S1PR1-dependent manner [195]. Since S1P has been shown to possess angiogenic and pro-inflammatory properties [196,197], a lymphangiogenic action is therefore hypothesized. Yoon *et al.* demonstrated that exogenous S1P induces lymphangiogenesis in both *in vitro* and *in vivo* systems. By treating human primary LECs with exogenous S1P and positive control VEGF-C, they found S1P induced migration and tube formation, but not proliferation of LECs. In vivo evidence was collected by the Matrigel plug assay, where S1P has been shown to act similarly to VEGF-C, inducing significant lymphangiogenesis. The molecular mechanism has been further addressed in studies using the genetic silencing of S1PR1 or S1PR3, where S1PR1, but not S1PR3, has been shown to be required in the lymphangiogenic action of S1P. S1P activates S1PR1; therefore, its coupled-Gi protein is activated to stimulate downstream phospholipase C to mobilize calcium to induce *in vitro* lymphangiogenesis. 

Given the role of Ang2 in lymphatic vessel development [54], Jan *et al.* further provided a link between S1P and Ang2 (Figure 1). Exogenous S1P treatment was shown to stimulate Ang2 exocytosis by either BECs or LECs. In agreement with Yoon *et al.*’s work, S1P acts through an S1P1/Gi/phospholipase C/Calcium signaling pathway to trigger Ang2 exocytosis [198]. Moreover, our group recently reported that SphK1-produced S1P promotes angiogenesis and lymphangiogenesis and facilitates breast cancer progression [199]. Because lymphatic metastasis is a major determinant for the staging and prognosis of breast cancer [200,201] and due to the importance of SphK1 in cancer progression as discussed above, we used an improved syngeneic breast cancer cell implantation method to examine the effect of SphK1 specific inhibitor, SK1-I, on tumor-induced lymphangiogenesis and cancer progression. In this model, we found inhibition of SphK1 decreases tumor growth, tumor burden, as well as lung metastasis. S1P levels and lymphangiogenesis in tumor are lowered by treatment with SphK1 inhibitor. We further demonstrated S1P acts similarly with Ang2 to exert angiogenic and lymphangiogenic effects on BECs and LECs. SphK1 inhibitor was shown to further abrogate the effect of Ang2. These results indicate that targeting S1P is a feasible therapeutic strategy for breast cancer and also shed light on the pathological effect of S1P in tumor-induced lymphangiogenesis.

To address the function of S1P in lymphatic system development [141], Pham *et al.* used an advanced animal model—SphK2 knockout mice with LEC-specific deletion of SphK1 (SphK^Δ^ mice). This sophisticated model was necessitated by the fact that SphK1 and SphK2 double knockout mice die in utero due to defects in blood vascular angiogensis and neurogenesis [202], whereas, SphK1-deficient or SphK2-deficient mice appeared morphologically and functionally normal [203]. Pham *et al.* found undetectable amounts of S1P in lymphatic fluid and no difference in blood S1P in their SphK^Δ^ mice compared to control mice. This ablation of lymphatic fluid S1P leads to aberrant lymphocyte trafficking and altered lymphatic vasculature. Along with this report, our group demonstrated the importance of S1P in the lymphatic system by examining Spns2-deficient mice [140]. We found aberrant lymphocyte trafficking and also a disrupted lymphatic vessel network in Spns2-deficient mice. Interestingly, Spns2-deficient mice showed decreased S1P in blood but increased concentrations in lymphatic fluid. Clearly, more work is needed to detangle the interrelationship between S1P production and exportation and the resultant impact on lymphatic system development.

**Figure 2:**
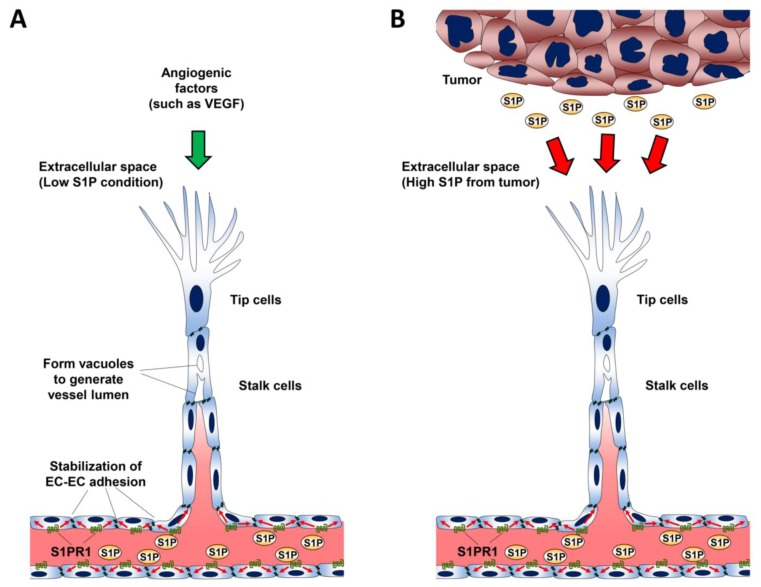
S1P and sprouting angiogenesis in normal development and in tumor-induced angiogenesis.

Recently, it has been reported that S1P in the blood circulation stimulates S1PR1 on the blood endothelial cells, which restricts sprouting angiogenesis, enhances the cell-to-cell adhesion, and stabilizes the vessels in the development process [204,205,206,207] (Figure 2A). Decreased expression of S1PR1 results in more aberrant sprouting, which actually interferes with vascular development and results in immature vascular networks in an S1PR1 knockout mouse model as well as in a model using morpholio-mediated knockdown of S1PR1 in a zebra fish [158,207]. Therefore, it is important to note that S1P regulates the vascular maturation in the development process by suppressing unnecessary sprouting and increasing the endothelial cell contact. Interestingly, it was recently shown that S1PR1 and S1PR2 cooperate to regulate the vascular development [207]. Moreover, Spns2, a newly identified S1P transporter, also cooperates with S1PR1 in this process [207], and as discussed above, we recently found that Spns2 has a role in lymphatic vessel network development as well [140]. Although roles for S1P in vessel sprouting in lymphangiogenesis and in lymphatic vessel stabilization are yet to be reported, S1P is expected to have a similar role in the development process of lymphatic vessels to its role in that of blood vessels considering that Pham *et al.* have reported that S1P secreted from lymphatic endothelial cells regulates lymphatic vessel maturation [141]. Furthermore, the role of S1P in tumor-induced angiogenesis and lymphangiogenesis needs to be investigated more precisely in this context; since S1P is provided not only from blood and endothelial cells, but also from tumors [199], and the contribution of S1P in the angiogenesis and lymphangiogenesis induced by tumors may be different from that which occurs in the normal vascular development processes (Figure 2B). In sum, S1P and S1PR1 regulate vascular development processes by restricting aberrant sprouting and stabilizing the vessels. Further investigation needs to be done especially in the cancer field.

These studies have firmly linked S1P to lymphangiogenesis in either molecular or biological aspects. Therefore, S1P might be a potential new addition to the growing list of lymphangiogenic factors secreted by LECs, inflammatory cells, and cancer cells to orchestrate the development and function of lymphatic system.

## 11. Conclusions

The lymphatic system contributes to important physiologic functions in fluid homeostasis, lipid transport, and in immune cell trafficking. Dysregulation of lymphangiogenesis provides a niche for uncontrolled inflammation and cancer progression. The field of lymphatic biology research remains young and needs more attention. Owing to the involvement of S1P in a wide range of physical and pathological process, the link between S1P and lymphangiogenesis provides important insight for further exploration of this field. Moreover, the emerging role of S1P as a lymphangiogenic lipid unveils the hidden regulatory mechanism which LECs, immune cells, or cancer cells utilize for promoting lymphangiogenesis in the context of inflammation and cancer. For therapeutic purposes, targeting S1P and S1P-metabolizing enzymes might be a feasible strategy. However, due to the massive complexity in the signaling pathways leading to lymphangiogenesis, more studies are still needed to establish an S1P pathway targeted therapy. 
